# Narrow-Band Light-Emitting Diodes (LEDs) Effects on Sunflower (*Helianthus annuus*) Sprouts with Remote Monitoring and Recording by Internet of Things Device

**DOI:** 10.3390/s22041503

**Published:** 2022-02-15

**Authors:** Thitiya Theparod, Supakorn Harnsoongnoen

**Affiliations:** 1 Department of Mathematics, Faculty of Science, Mahasarakham University, Kantarawichai District, Maha Sarakham 44150, Thailand; thitiya.t@msu.ac.th; 2 The Biomimicry for Sustainable Agriculture, Health, Environment and Energy Research Unit, Department of Physics, Faculty of Science, Mahasarakham University, Kantarawichai District, Maha Sarakham 44150, Thailand

**Keywords:** narrow-band light-emitting diodes (LEDs), sunflower (*Helianthus annuus*) sprouts, Internet of Things (IoTs) device, germination, sigmoidal growth curves

## Abstract

Previous studies have demonstrated that light quality critically affects plant development and growth; however, the response depends upon the plant species. This research aims to examine the effects of different light wavelengths on sunflower (*Helianthus annuus*) sprouts that were stimulated during the night. Natural light and narrow-band light-emitting diodes (LEDs) were used for an analysis of sunflower sprouts grown under full light and specific light wavelengths. Sunflower seeds were germinated under different light spectra including red, blue, white, and natural light. Luminosity, temperature, and humidity sensors were installed in the plant nursery and remotely monitored and recorded by an Internet of Things (IoT) device. The experiment examined seed germination for seven days. The results showed that the red light had the most influence on sunflower seed germination, while the natural light had the most influence on the increase in the root and hypocotyl lengths.

## 1. Introduction

Light is an important factor that affects photosynthesis in plants, because it is the main source of energy that will continue physiological processes and has a great influence on plant development in which particular responses of plants could be affected by specific wavelengths [1]. In particularly, stem growth inhibition, leaf expansion, phototropic growth, and accumulation of anthocyanin pigments are controlled by blue light (i.e., wavelength of 460–500 nm). Red light (i.e., wavelength of 620–720 nm) regulates multiple responses including germination, chloroplast function, stem, and petioles. Far-red light (i.e., wavelengths of 700–800 nm) promotes growth of different parts of plants such as the size of leaves, stem length, and the height of plants [2]. However, plant growth can also vary depending upon plant species and light spectrum as well as other factors such as temperature, humidity, and irrigation. The impact of light quality is complex, and mixed results have often been reported [3,4,5]. Several studies have shown that the LED light sources have an effect on many plants such as pea [6], cotton [7], rice [8], vegetables [9], broccoli [10,11], lettuce [12], tomato [13], cowpea [14], and *oncidium* orchids [15]. The number of studies on the benefits of LED lighting on plant growth and development have increased over the years because of the fact that LEDs have many advantages over other conventional artificial light sources such as smaller size, cheaper price, longer lifetime, and durability [14,16]. Moreover, LEDs can be controlled for specific wavelength and narrow bandwidth. This allows the wavelength to be matched to plant photoreceptors. The light quality, which refers to the spectral distribution of light, color, or wavelength reaching the surface, strongly influences plant development, physiology, and metabolism [17]. The ability to control the quality of light, such as intensity, enables us to improve the quality of plant growth, development, and nutritional quality, which is ideal for plant lighting designs. Therefore, LED devices have become an important component of smart precision lighting for urban and landscape closed-controlled horticultural environments [18], and it is a currently growing business that is spreading around the world [19].

Sunflowers (*Helianthus annuus*) are one of the most important germinated seeds, because they are a source of protein, vitamins, and minerals with less calories [20]. They can be germinated without soil or sunlight [21]. Sunflower is an important raw material in food production and medicines [22]. It also contains various important chemical elements. It can be used to help treat many serious or life-threatening diseases [23]. Sunflower seeds produce large sprouts, succulent white stems, and the leaves are tasty and rich in chlorophyll. In addition, sunflower sprouts also contain components with biological activity and are rich in vitamins, minerals, amino acids, fatty acids, vitamins (especially E), selenium, copper, zinc, folate, iron, and fiber [24].

The Internet of Things (IoTs) can monitor and control processes to increase productivity and the sustainability of production processes [25,26,27]. IoT systems can be used to monitor and interact in real time with the environment by using sensors that can communicate and transmit data to each other or to a control room over the internet [28]. Precision agricultural (PA) technology brings IoT to more applications, it is used as a technology for automated data sensing, data collection, and yield monitoring [29,30] in fields such as seed testing [31], sugarcane [32], poultry farm [33], Brassica chinensis [34], soil [35], greenhouses [36], agro-industrial and environmental disciplines [37]. However, there have been no studies and analyses of narrow-band LED effects on sunflower (*Helianthus annuus*) sprouts that were stimulated during the night. Therefore, the objective of this work was to investigate the influence of narrow-band red, blue, and white light on seedling quality and the growth of sunflower plants and sprouts using monitoring with IoT devices. Sunflowers were chosen as model plants because of the characteristics of their distinct growth stages and how sensitive the plant responds to light.

## 2. Materials and Methods

### 2.1. Sample Preparation

**Figure 1:**
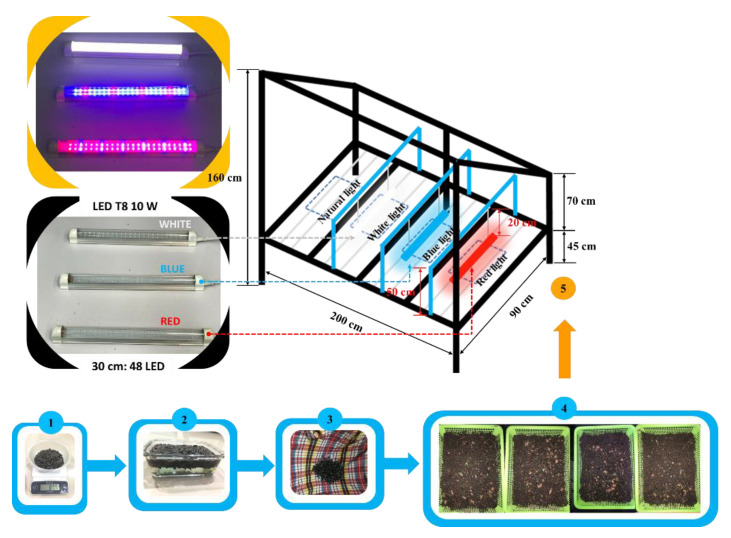
Process of seedling preparation: (**1**) selecting sunflower seeds; (**2**) soaking sunflower seeds in water; (**3**) wrapping the seeds in a towel; (**4**) planting the seeds in the nursery tray; (**5**) installing a nursery tray in the plant nursery.

The process of sample preparation is shown in Figure 1. We selected completely black and plump seeds of sunflower that were uniform in size, weighing a total of 87.7 g, and their surfaces were sterilized with chemical reagents. The sterile seeds were washed with distilled water until the neutral pH value was met. After sterilization, seeds were submerged in water for 8 h. The seeds were cultivated by wrapping them in a wet cloth for 20 h to accelerate the germination of the radical roots of the seeds. After germination, 120 sunflower seeds were selected to plant in four trays, 30 seeds each. The seedling trays were cleaned and sterilized thoroughly before sowing and culturing. Cultivated soil consisted of soil (fine soil with neutral PH), coconut coir, and black husk in a mixing ratio of 2:1:1.

### 2.2. Treatments and Experimental Setup

**Figure 2:**
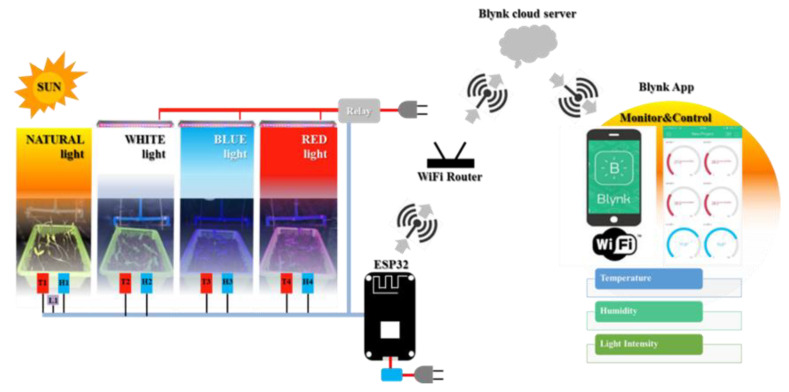
The proposed structure of the system architecture.

The proposed structure of the system architecture is shown in Figure 2. The system was divided into three parts: the plant nursery and the sensor device; the communication unit consisted of an ESP32 microcontroller with built-in Wi-Fi; an Android/iOS-based mobile user interface. The plant nursery consisted of seed experiments under 4 different treatments of light: (1) natural light (control); (2) red light; (3) blue light; (4) white light. The red, blue, and white light sources were provided by an LED lamp, 30 cm above the trays. Each LED color source had a power of 10 W (input voltage: AC 85–240 V/50–60 Hz; style: T8 (Double line); cost: USD 17.85 (red), USD 17.85 (blue), and USD 15.97 (white); brand: Haolin, China. The LED lights only worked at night (6:00 p.m.–6:00 a.m.). The plant nursery was made of a steel box with a width of 90 cm, length of 200 cm, and height of 165 cm as shown in Figure 1. The natural light that the sunflower sprouts received was sunlight that shined on the open corridors of the building. The light intensity was attenuated using a black mesh, and the intensity was recorded via the IoT system. Sunflower sprouts in all trays were arranged to provide equal exposure to natural light during the day. Temperature/humidity sensors (DHT11) were also installed in every treatment. The area receiving natural light was equipped with an additional light sensor (BH1750FVI). The ESP32 (model ESP32-WROOM-32 from ESPRESSIF, China) with a dual-core structure, and a built-in Wi-Fi module was used for environmental monitoring. The LEDs were powered on and off with a 220 VAC power supply via a relay controlled by the ESP32 and powered by a 5 VDC adapter. Data were collected using a no memory element system and was directly saved in the Blynk cloud server via the mobile interface. This system allows users to retrieve the data with a registered email address. The Internet connection status was always checked before transferring data. If the internet was disconnected, temporary non-transferable data sets were stored to ensure the integrity of the data; when connecting to the internet successfully, these data sets were automatically sent to the cloud server. The temperature, humidity, and brightness data were recorded and calculated, on average, every 1 h for a period of 7 days. The germinated seeds were watered twice a day: morning and evening in equal amounts for each tray. The monitor and the control front panel was developed as an iOS- and Android-based mobile user interface. Blynk is an IoT platform used to measure, record, and display data. Information, such as temperature, humidity, and light, were stored in the Blynk cloud server, and the control systems could easily be accessed at any time.

**Figure 3:**
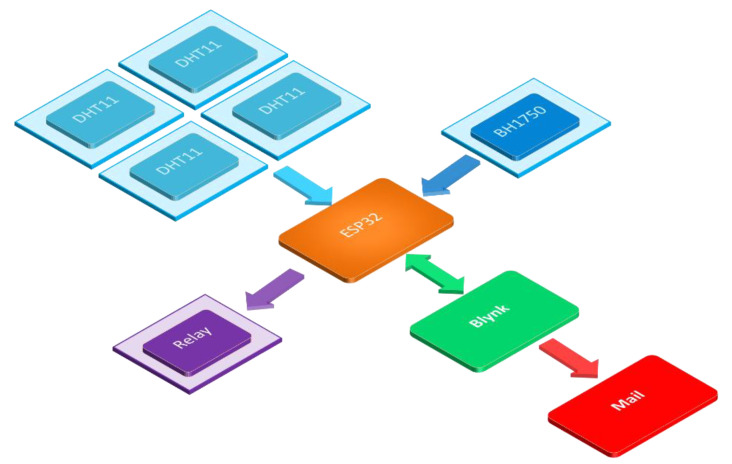
Data transmission diagram of the plant nursery control system.

The transmission of information of the nursery control system is shown in Figure 3. The ESP32 microcontroller communicates with the Blynk app to receive commands and to send data between them. The ESP32 microcontroller sends a signal to control the LED light on/off switch via a relay which commutated through the Blynk app and receives measurements of light luminance, temperature, and humidity from the sensors. All information was sent to the Blynk app. Once the Blynk app received the measurement data, the app displayed the measurement results on the mobile phone screen and sent the measurement data via email.

**Figure 4:**
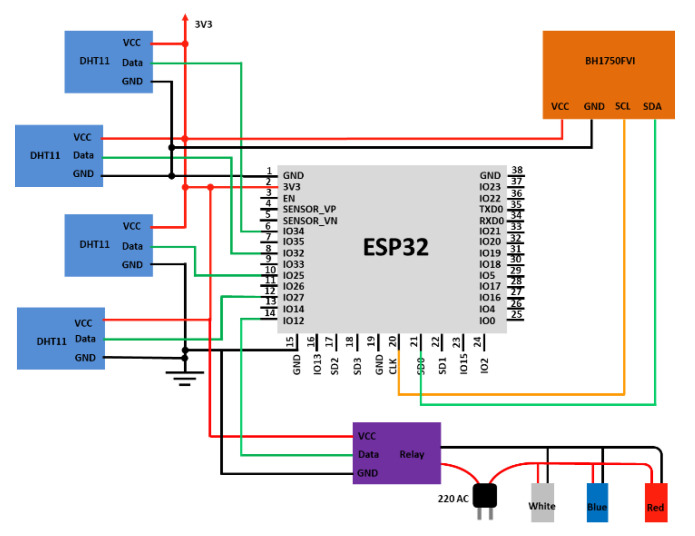
Circuit diagram.

Figure 4 shows the details of the circuit diagram for an environmental sensing system. The electronic components of the device consisted of microcontroller (ESP32), relay, temperature and humidity sensor (DHT11), and ambient light sensor (BH1750FVI). We used 10 pins to connect all sensors with the ESP32 board. The pins were 3.3 V pin, 2 GND pins, 5 multiplexed IO pins, CLK pin, and SD0 pin. There are still many pins that were not in use, which could be useful for our system improvements and scaling in the future using the same board. One relay and four sensors of DHT11 digital temperature and humidity were used. The relay control pin was connected to the ESP32′s IO12 pin in order to control the LEDs on/off switch. The data pin of the sensors was connected to IO25, IO27, IO32, and IO34 pin of the ESP32 board. The exclusive digital signal acquisition process for sensing humidity and temperature of the DHT11 sensor ensured high reliability and excellent long-term stability. The humidity accuracy, temperature accuracy, and resolution were ±5% RH, ±2 °C, and 1, respectively. The BH1750FVI device, a digital ambient light sensor for the I2C bus interface, was selected to use in this study. The VCC pin of the sensor was connected to the 3v3 pin, while the GND pin was connected to GND pin on the ESP32 board. The SCL (serial clock) pin of the sensor was connected to the CLK pin and the SDA (serial data) pin of the sensor was connected to the SD0 pin of the ESP32. These four connections were part of a standard i2c hardware connection to the I2C sensor.

**Figure 5:**
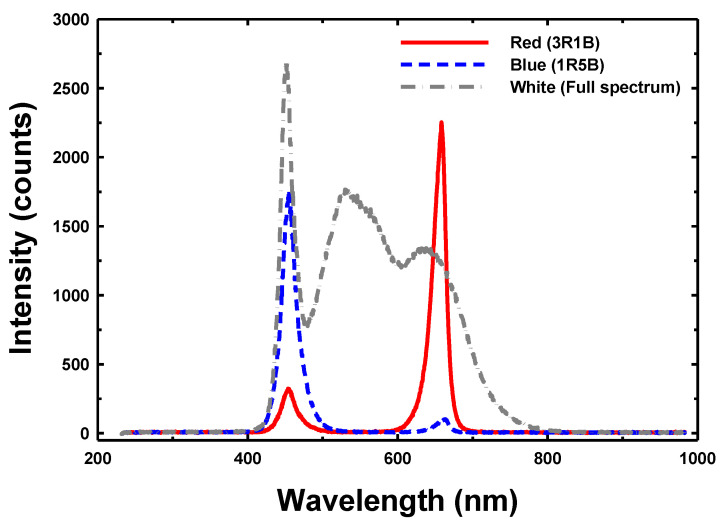
Light spectra of the red (3R1B), blue (1R5B) and white (Full spectrum) lighting at plant nursery.

In this study, we installed the LED light close to the nursery tray. Therefore, we used LED tubes T8 consisting of LEDs with primary optics attached. The LED tubes T8 had a wide light distribution pattern to examine the effects of different light wavelength on sunflower (*Helianthus annuus*) sprouts that were stimulated during the night. The light spectra of LEDs were monitored using an Ocean Optics HR2000 spectrometer interfaced and connected to a personal computer via a USB port. The relative spectra of the light treatments are shown in Figure 5. Red (3R1B) light: 75% red LED light with a wavelength of 660 nm and 25% blue light with a wavelength of 460 nm; blue (1R5B) light: 16.7% red LED light with a wavelength of 660 nm and 83.3% blue light with a wavelength of 460 nm; white (full-spectrum) light with a wavelength of 420–780 nm. The length of the LED lamps was 30 cm with a power of 10 W. The sun was the source of natural light (control), which had a different light intensity within the nursery at different times of the day.

### 2.3. Statistical Analysis

All data were examined for normality and homogeneity of variance using the Shapiro–Wilk test and the Bartlett test, respectively. The measurements were evaluated for significance by a Kruskal–Wallis H test that was used to analyze the data that did not meet the normal distribution and/or homogeneity of variance. All statistical analyses were conducted using R version 4.0.5.

## 3. Results and Discussion

### 3.1. Seed Germination

**Figure 6:**
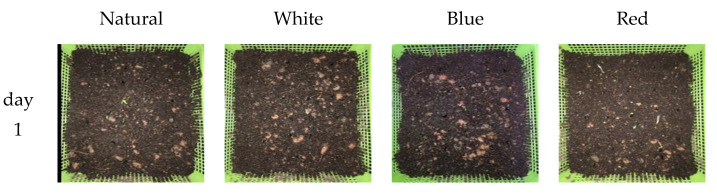

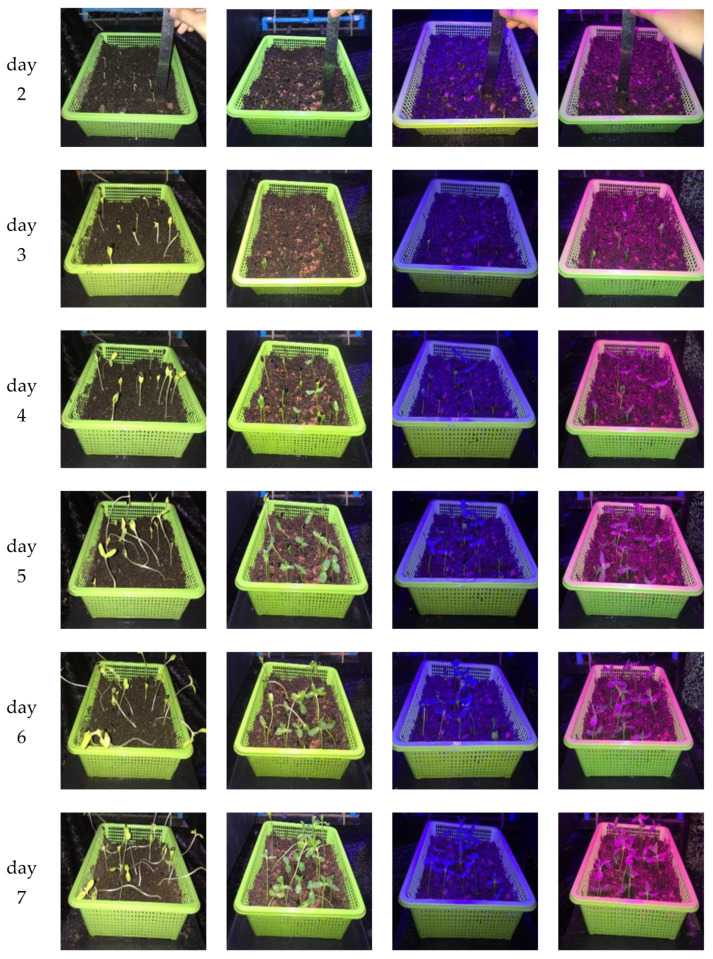
Sunflower seedlings treated under natural, white, red, and blue lights for 7 days.

**Figure 7:**
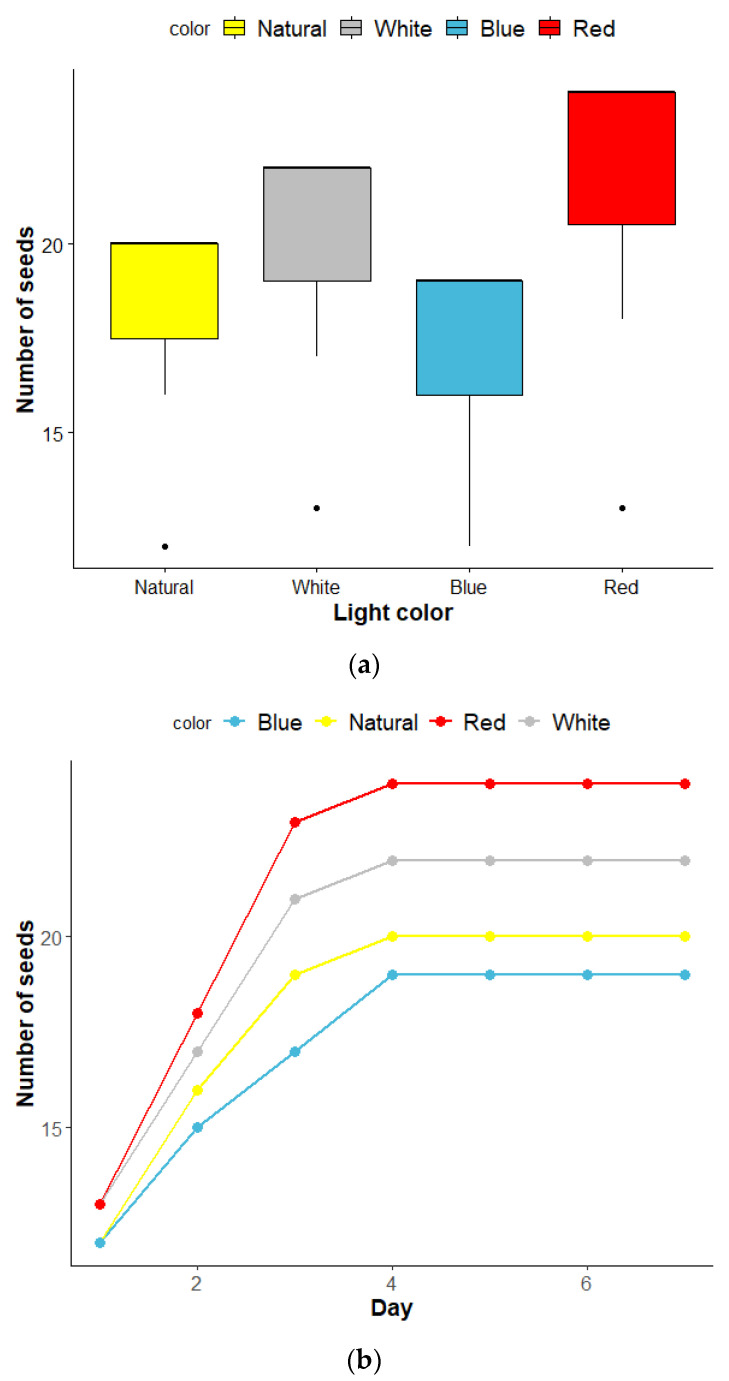
Germination number of seeds under red (3R1B), blue (1R5B), white (full spectrum), and natural light treatments for each plant nursery: (**a**) comparison between treatments; (**b**) number of germinated seeds per day.

Figure 6 represents sunflower seedlings treated under four different light sources, which were natural, white, red, and blue light LEDs for 7 days. Sunflower seed germination was significantly affected by the light spectrum ($\chi^{2}$ = 7.94, *p* = 0.0472). Figure 7a,b, respectively, show a comparison of the number of germinated seeds between treatments and the number of seeds germinated each day until the end of the process. During the first few days of germination, it was observed that the four different light sources resulted in different germination success numbers: 24 for red, 22 for white, 20 for natural, and 19 for blue light, which accounted for germination percentages of 80%, 73.33%, 66.67%, and 63.33%, respectively.

From the 4th day onwards, the numbers were unchanged. As a result, we calculated the germination indices; the results ranked from highest the lowest were 3.43 (red), 3.14 (white), 2.86 (natural), and 2.71 (blue). This indicates that red light provided the highest ability for germination of sunflower seeds, while blue light was the lowest. Our results are in line with the literature but for different plant species, i.e., Cucumis callosus seeds, Pinus virginiana Mill seeds, and M. aegyptia. Moreover, we observed that natural light had a germination success number close to that of white light. This may be because the natural light has a color similarly to white. The germination of seeds exposed to different light colors corresponding to the number of germination days can be expressed as an exponential rise function as shown in Table 1.

### 3.2. Temperature, Humidity, and Luminosity Factors

**Figure 8:**
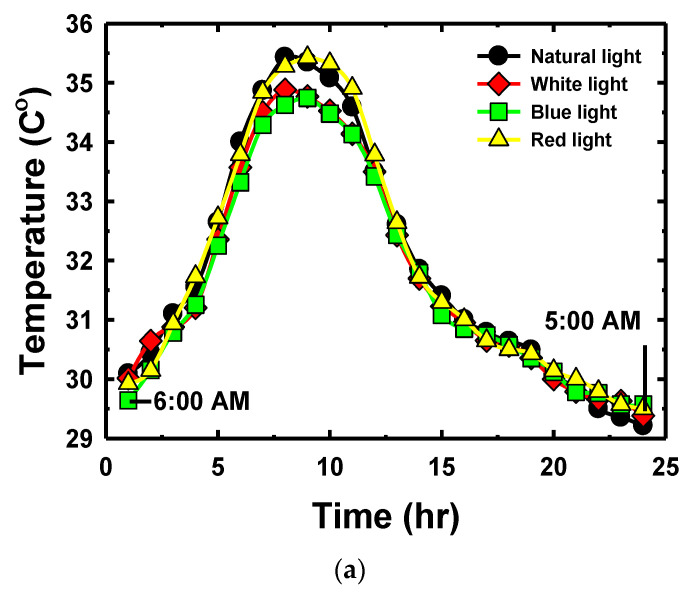

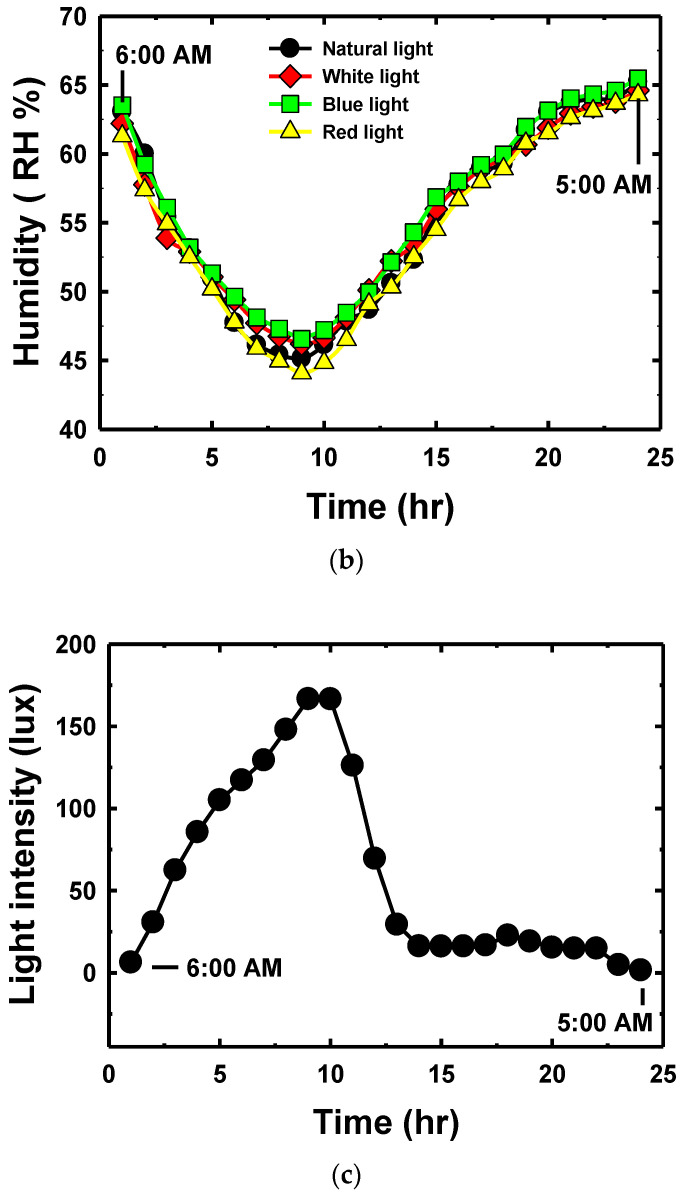
Environmental factors (**a**) temperature; (**b**) humidity; (**c**) light intensity.

Figure 8a shows the mean temperature of each day for seven days in the nursery trays under four bands of light from 6:00 a.m. to 5:59 a.m. The mean temperature of the nursery trays under the four bands of light in the range of 5:00 a.m. to 6:00 a.m. (the connection point between day and night) was 29.67 °C. The mean temperature between 9:00 a.m. and 3:00 a.m. was 33.73 °C, temperature differences in the four culture trays during this period was ±0.28 °C. The mean of temperature at noon of the four trays was 35.07 °C; the trays on the left (natural light) and right sides (red light) of the nursery were slightly higher than the trays inside (white and blue light) which has a difference value in the range of ±0.37 °C. Figure 8b shows the mean humidity of each day for seven days in the culture trays under the four bands of light from 6:00 a.m. to 5:59 a.m. It shows that the mean humidity of the culture trays under the four light bands in the range of 5:00 a.m. to 6:00 a.m. was 63.71%. The mean humidity from 9:00 a.m. to 3:00 a.m. was 48.50%, and the humidity differences in the four culture trays during this period was ±0.81%. The mean temperature of all trays was similar and had a similar variation pattern. The measured humidity was inversely proportional to the temperature. Figure 8c shows the luminance of the light inside of the plant nursery. The luminance of the light at 6:00 a.m. and at 5:00 a.m. was 3.34 and 3.14 lux, respectively. From 2:00 p.m. to 3:00 p.m., the average luminance value was 171.26 lux. Then, the luminosity decreased until 5:00 p.m. when it reached a value of 31.33 lux. The average of luminance value between 7:00 p.m. and 3:00 a.m. was 16.95 lux. The average temperature, humidity, and luminosity for each time period during the day and night in the nursery are shown in Table 2. During the day, there were four periods, each with a duration of three hours, starting from 6 am. to 6 pm. At night, we considered only one period (a total of 12 h). The temperature in the nursery during the day ranged from 30.40 to 34.92 °C, with an average of 33.03 °C. While the humidity in the nursery during the day ranged from 46.18% to 58.73%, with an average of 50.88%. From the measurement results, it was found that the temperature at the natural light location and the LED light installation location were similar with a slight difference (less than 1 °C). The results clearly showed that the light that came from the LED tube did not generate enough heat to affect the DHT11 sensors and sunflower sprouts.

### 3.3. Length of Hypocotyl

The hypocotyl was stimulated by four different light sources during the night. We found that there was no significant difference in the length of the hypocotyl ($\chi^{2}\;$= 3.68, *p* = 0.298). However, we observed that the germinated seeds treated under the four narrow-band LEDs had different stem lengths and could be classified into different size groups. For better analysis, we divided them into three groups; short (group A), medium (group B), and long (group C) (see Table 3).

**Figure 9:**
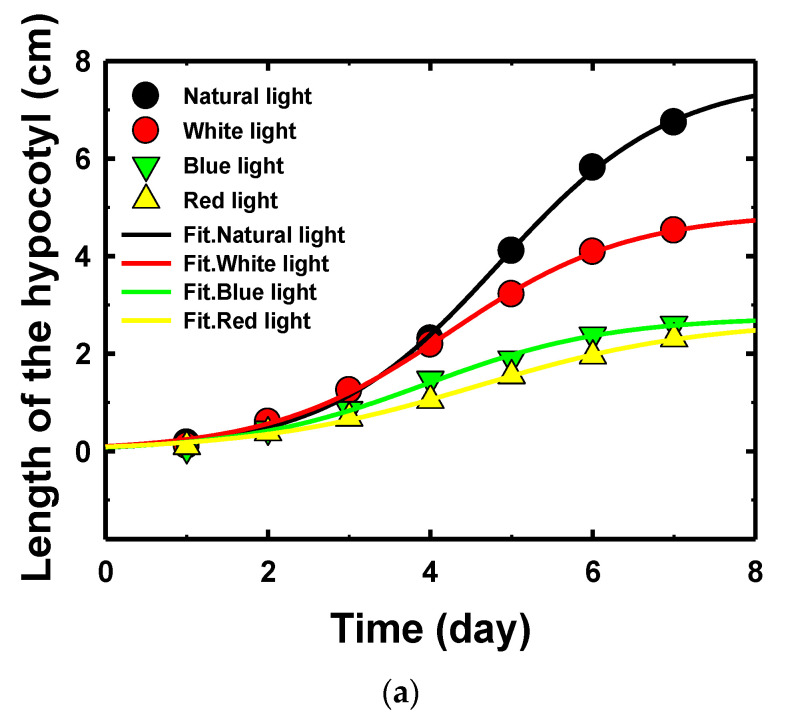

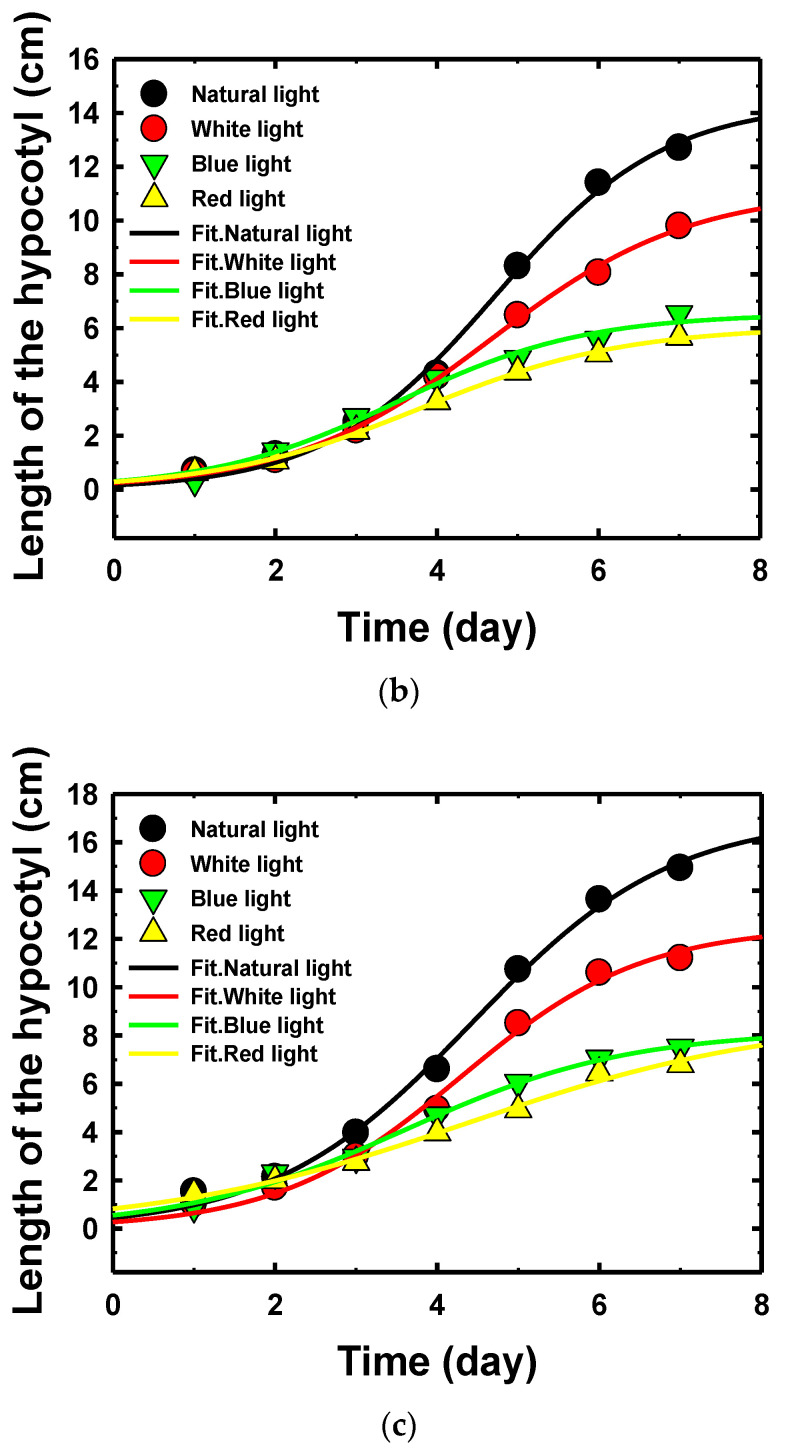
Comparison of the effect of four light spectra between length groups of hypocotyls: (**a**) short (group A); (**b**) medium (group B); (**c**) long (group C).

The comparison between the lengths of stems of sunflower seedling treated under four light spectra corresponding to days of germination process is shown in Figure 9a–c, respectively, for the length groups A, B, and C. The results show a trend in which the lengths of stems in every treatment were similar during the first few days of germinated seeds, which shows that the color of light did not have an effect on the germinated seeds at early stages. However, from the 4th day onwards, the results displayed larger differences in stem length between each treatment. Group C was observed to display growth saturation in sprouts under all four colors of light. In groups A and B, a slight slowing of growth was observed in sprouts grown under natural light, whereas there was no growth saturation under the light-emitting diode light. It is possible groups A and B under light-emitting diode light might grow for a longer period.

The mean stem lengths of groups under natural light, white light, blue light and the red light were 2.98, 2.82, 1.38, and 1.14 cm, respectively. Group B, natural light, white light, blue light, and red light had mean stem lengths of 5.88, 4.63, 3.65, and 3.17 cm, respectively. The mean of lengths of stems in group C for natural light, white light, blue light and red light was 7.64, 5.85, 4.49, and 4.04 cm, respectively. From the study, it was found that sunflower seeds grown under natural light in dark condition during the night had whiter and longer hypocotyls as compared to sunflower seeds that were illuminated by LED lamps at night. This is in line with tartary buckwheat [38], soybean [39], dolichos, and cowpea sprouts that are stimulated by FLs [40], which show that the sprouts grown in the dark show etiolation phenotype, with white and long hypocotyls.

**Figure 10:**
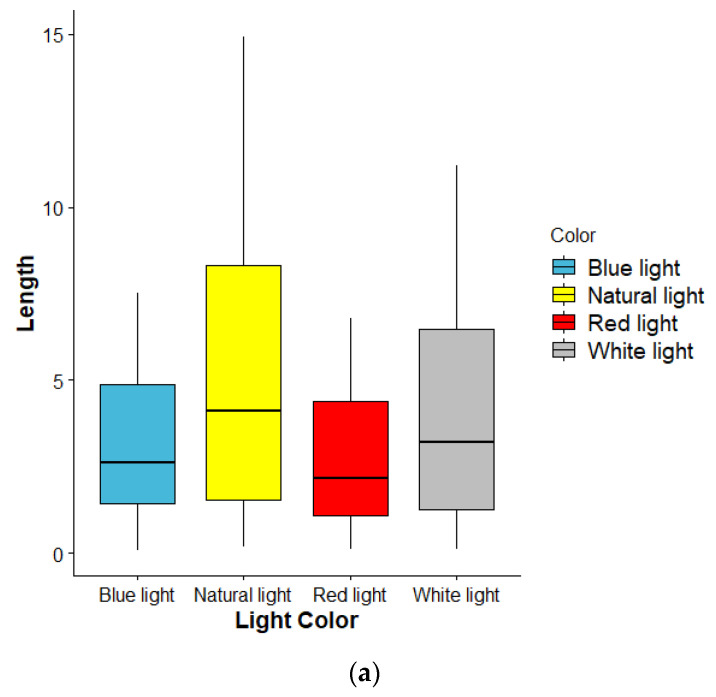

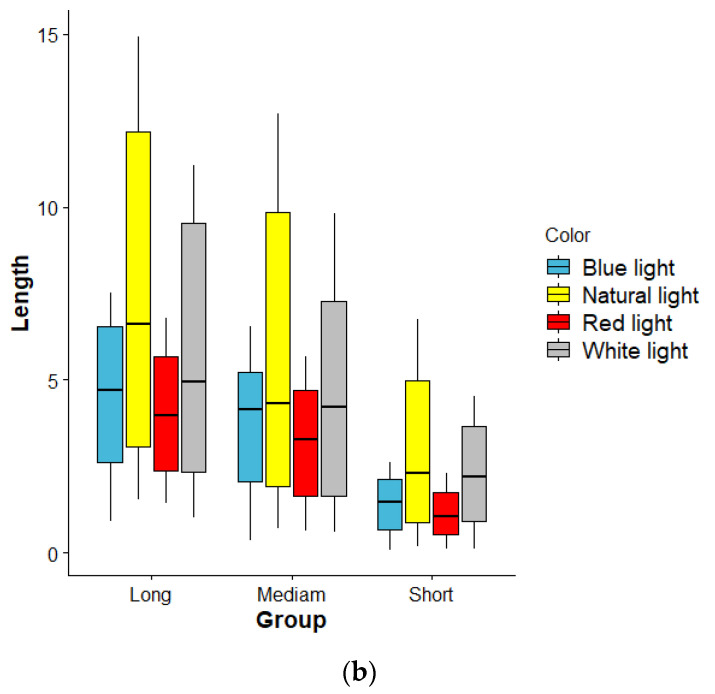
Comparison of the effects of the four light spectra on the length of hypocotyls (**a**) and comparison of the effect between length groups (**b**).

Vice versa, the sprouts grown under light, the shorten hypocotyl of light-grown sprouts may be due to the inactivation of constitutive photomorphogenic 1 (COP1), which is an inhibitor for photomorphogenesis and stability of long hypocotyl 5 (HY5), and long hypocotyl 5 homolog (HYH). It may also be derived from plant hormones, such as gibberellins, and ethylene may contribute to the light-controlled hypocotyl elongation [41,42]. Comparing red, blue, and white LED lights, LED white light alone could significantly increase hypocotyl length of sunflower sprouts when compared with red and blue light. This may be because white light has a spectrum closer to that of natural light than red and blue lights. The blue light makes hypocotyl longer than red; this is likely because blue light has a greater effect on hypocotyl stretching than red light. The above results contradict with previous research results [43], and this may be due to the difference in the period of time that the sunflower sprouts were exposed to light with different light intensities in the study. Moreover, we found that the sunflower sprouts grown under natural light had bending shapes, whereas sunflower seeds grown under light from light-emitting diodes had straight stem shapes. Probably because sunflower sprouts in the natural light group were not equally exposed to natural light in same the intensity and direction. Unlike sunflower seedlings in the group that received LED lights during the night, which has a constant direction and intensity, resulting in more straight shapes of sunflower sprouts. The lengths of the hypocotyl with respect to days of germination process can be expressed as a Sigmoid function of germination date as shown in Table 4. Figure 10a shows the comparison among light groups, and it is observed that the lengths of stems under natural light were higher than those under the other three light spectra. Moreover, the same behavior was also observed when comparing by length group (Figure 10b).

### 3.4. Summary of the Effects of LED Light on the Growth and Nutrients of Sprouts and Plants

LEDs are one of the most important artificial light sources for sprouts and plants because they can be applied to control growth, formation of phytochemical compounds, and antioxidants of sprouts and plants. There are many advantages, such as low cost, light weight, low power consumption, and high durability, and their light range and the duration of lighting can be controlled. Numerous studies have highlighted the effect of LEDs on control growth, formation of phytochemical compounds, and antioxidants of sprouts and plants. The details of effects of LED light on the control growth, formation of phytochemical compounds and antioxidants of sprouts and plants are summarized in Table 5.

## 4. Conclusions

Sunflower (*Helianthus annuus*) is an important raw material in the production of food, medicine and biological activities. The use of a favorable lighting environment for seeds germination has attracted much attention in the last decade. We used sensor technology to measure the environment and studied the impact on sunflower sprout through IoT system. The proposed IoT system demonstrates the simplicity and low cost of measuring, controlling and recording important data in studying the narrow-band light emitting diodes (LEDs) effects on Sunflower sprouts. The results of our study indicated that the narrow-band LEDs in the red light region was the most effective for sunflower seed germination as compared to other light colors, blue, white, natural lights. Sunflower seeds grown under natural light have the longest hypocotyl. Moreover, we found that germination dynamics and length of hypocotyl could be described by sigmoidal growth curves. The proposed system can be improved further in the future by increasing the number of sensors and other sensor types to get more complete and comprehensive information to enhance the efficiency of water, light, gas, and plant nutrient management in precision agriculture in the future.

## Figures and Tables

**Table 1:** Exponential rise to maximum with the germinated seed with the time.

Light	Exponential Rise Function (Seed)	*R* ^2^
Natural	Y = 20.237 × (1 − exp(−0.864X))	0.9861
White	Y = 22.327 × (1 − exp(−0.825X))	0.9741
Blue	Y = 19.018 × (1 − exp(−0.887X))	0.9533
Red	Y = 24.603 × (1 − exp(−0.744X))	0.9738

**Table 2:** Temperature, humidity, and luminosity in the day and nighttime.

Time		Temperature (°C)	Humidity (RH %)	Light Intensity (lux)
Day	6:00 a.m.–8:59 a.m.	30.40 ± 0.47	58.73 ± 3.32	33.10 ± 28.11
	9:00 a.m.–11: 59 p.m.	32.53 ± 0.98	50.81 ± 1.93	102.56 ± 15.91
	12:00 p.m.–2: 59 p.m.	34.92 ± 0.38	46.18 ± 1.19	147.93 ± 18.63
	3:00 p.m.–5: 59 p.m.	34.28 ± 0.64	47.80 ± 1.59	120.71 ± 48.75
Night	6:00 p.m.–6: 00 a.m.	30.56 ± 0.92	59.63 ± 4.36	15.59 ± 7.19

**Table 3:** Criteria dividing stem lengths into 3 groups.

Light	Small (cm)	Medium (cm)	Long (cm)
Natural	≤0.6	0.6 < x ≤ 1.2	1.2 < x ≤ 1.8
White	≤0.4	0.4 < x ≤ 0.8	0.8 < x ≤ 1.2
Blue	≤0.4	0.4 < x ≤ 0.8	0.8 < x ≤ 1.2
Red	≤0.5	0.5 < x ≤ 1.0	1.0 < x ≤ 1.5

**Table 4:** Sigmoid function of the length of hypocotyl.

Groups	Light	Sigmoid Function (cm)	*R* ^2^
Small	Natural	Y = 7.659/(1 + exp(−(x − 4.838)/1.059))	0.9994
	White	Y = 4.887/(1 + exp(−(x − 4.235)/1.100))	0.9987
	Blue	Y = 2.751/(1 + exp(−(x − 3.944)/1.132))	0.9951
	Red	Y = 2.683/(1 + exp(−(x − 4.583)/1.369))	0.9975
Middle	Natural	Y = 14.390/(1 + exp(−(x − 4.719)/1.052))	0.9957
	White	Y = 11.139/(1 + exp(−(x − 4.661)/1.237))	0.9983
	Blue	Y = 6.539/(1 + exp(−(x − 3.516)/1.167))	0.9866
	Red	Y = 6.052/(1 + exp(−(x − 3.800)/1.267))	0.9986
High	Natural	Y = 17.055/(1 + exp(−(x − 4.431)/1.228))	0.9960
	White	Y = 12.529/(1 + exp(−(x − 4.284)/1.128))	0.9929
	Blue	Y = 8.204/(1 + exp(−(x − 3.609)/1.374))	0.9939
	Red	Y = 8.766/(1 + exp(−(x − 4.380)/1.936))	0.9927

**Table 5:** Effect of light and light-emitting diodes on growth of sprout and plant.

References	Sprouts/Plants	Light Sources	Discovered Activity
[3]	Baby leaf lettuce (*Lactuca sativa* L.)	UV-A, blue (B), green (G), red (R), and far-red (FR) light-emitting diodes (LEDs)	B increased anthocyanins accumulation and carotenoids concentration. UV-A increased anthocyanins accumulation. R increase phenolics concentration. FR light increase biomass.
[4]	Romaine lettuce (*Lactuca sativa* L.)	Light intensity (100–800 μmol m^−2^ s^−1^)	400–600 μmol m^−2^ s^−1^ is a recommendable light intensity for production of lettuce cultivar Lvling.
[5]	Tomato (*Solanum lycopersicum* L.), oriental plane (*Platanus orientalis* L.)	RGB (red 33%, green 33%, bblue 33%) and RB (red 66%, blue 33%) light-emitting diodes (LEDs) and white light (WL)	The plant height, plant biomass and leaf area were significantly reduced by RGB and RB compared to WL reduced.
[6]	Pea seedlings	Red (625–630 nm) and blue (465–470 nm) LED lights	Red light supports the expression of important β-carotene and antioxidant activity for nutrition and health benefits. Blue light increased seedling weight and chlorophyll induction of irradiated butterfly pea seedlings.
[7]	Cotton (*Gossypium hirsutum* L.)	Fluorescent lamp, monochromatic blue LED (B), three blue and red LED mixtures (B:R = 3:1, 1:1, 1:3) and monochromatic red LED (R)	Blue LED light increased chlorophyll content, leaf thickness, palisade tissue length, leaf and stomata area. Red LED light increased root activity, sucrose, starch and soluble sugar contents. The blue and red LEDs (B: R = 1:1) are the highest suitable lighting for growing of upland cotton platelets in vitro.
[8]	Rice (*Oryza sativa* L.)	The LED fixture could emit blue, red, blue:red (B:R) = 1:1, B:R = 1:3, B:R = 1:8, yellow, and green light	Blue (B) light is the most appropriate light for rice tissue culture plantlets and a blue:red (B:R) = 1:1 LED light facilitated the cultivation of robust rice seedlings.
[9]	Seedlings of 18 vegetable genotypes	Red and blue light-emitting diodes	The RB-LED lighting affects many aspects of plant development during and after germination testing the genotypes of 18 vegetables.
[10]	Broccoli (*Brassica oleacea var. italica*)	Fluorescent/incandescent light; 5% blue (442 to 452 nm)/95% red (622 to 632 nm); 5% blue/85% red/10% green (525 to 535 nm); 20% blue/80% red; and 20% blue/70% red/10% green	The fluorescent/incandescent light treatment induced to significantly lower concentrations of most metabolites measured in the sprouting broccoli tissue. The blue light wavelengths from LEDs can be stimulated of primary and secondary metabolite biosynthesis.
[11]	Broccoli (*Brassica oleacea var. italica*)	Short-duration blue light LEDs	Short-duration blue light increase important phytochemical compounds influencing the nutritional value of broccoli microgreens.
[12]	Lettuce (*Lactuca sativa* L.)	Blue, green and red light LEDs, and white fluorescent light	The energy transmitted by green light is effective in balancing biomass production and secondary metabolite production involved in plant protection.
[13]	Tomato (*Solanum lycopersicum* L.) and cucumber (*Cucumis sativus*)	HPS sodium lamps and LEDs	Tomatoes and cucumbers grown under LED lamps have a higher chlorophyll index than is grown under HPS lamps and without any light.
[14]	Cowpea (*Vigna unguiculata* L.)	Blue, green and red light LEDs	Overhead LED lights make the intracanopy-grown cowpea grow lead to increased productivity and biomass.
[15]	*Oncidium* orchids	Fluorescent lamps (FL), red light emitting diodes (LEDs) (RR), blue LEDs (BB), yellow LEDs (YY) and green LEDs (GG).	The spectrum of red and blue LEDs are efficient for use as excellent energy for in vitro seedling growth. Red LEDs promoted protocorm-like bodies (PLBs) induction in *Oncidium*, and that blue LEDs promoted differentiation.
[16]	Sprouts and microgreens	High-pressure sodium lamps (HPSs), fluorescent lamps (FLs) and light-emitting diodes (LEDs)	LEDs are a promising lighting for improving the nutrient quality in sprouts and microgreens. Sprouts and microgreens culturing under LED sources could regulate the growth, the phytochemical compound content and antioxidant capacity of sprouts and microgreens.
[17]	Lettuce (*Lactuca sativa* L.)	Fluorescent lamps (FL), red LEDs, blue LEDs, and d blue + red (BR) LEDs	The antioxidant activities of seedlings treated with blue-containing LED lights were higher than that under FL and red light. Seedlings treated with blue light promoted the growth of lettuce plants after transplanting.
This work	Sunflower (*Helianthus annuus*)	Natural light, white, red and blue LEDs	The narrow-band LEDs in the red light region was the most effective for sunflower seed germination as compared to other light colors, blue, white and natural. lights. Sunflower seeds grown under natural light have the longest hypocotyl.

## Data Availability

Not applicable.
